# A phase Ib study evaluating the c-MET inhibitor INC280 (capmatinib) in combination with bevacizumab in patients with high-grade glioma

**DOI:** 10.1093/noajnl/vdae220

**Published:** 2024-12-11

**Authors:** Gerald S Falchook, James D Battiste, Amandeep Kalra, Mythili Shastry, Lindsey Finney, Susan J Hoekstra, Meredith G Shih, Kent C Shih

**Affiliations:** Drug Development, Sarah Cannon Research Institute at HealthOne, Denver, CO, USA; Neuro-Oncology, Oklahoma University Health, Oklahoma City, OK, USA; Medical Oncology, HCA Midwest Kansas City, Kansas City, KS, USA; Drug Development, Sarah Cannon Research Institute, Nashville, TN, USA; Drug Development, Sarah Cannon Research Institute, Nashville, TN, USA; Drug Development, Sarah Cannon Research Institute, Nashville, TN, USA; Greco Hainsworth Centers for Research at Tennessee Oncology, Nashville, TN, USA; Greco Hainsworth Centers for Research at Tennessee Oncology, Nashville, TN, USA; Drug Development, Sarah Cannon Research Institute, Nashville, TN, USA

**Keywords:** INC280, capmatinib, glioma, bevacizumab, c-MET inhibitor

## Abstract

**Background:**

To improve survival in patients with high-grade glioma, INC280 (capmatinib) a highly selective and potent oral inhibitor of the MET receptor with robust central nervous system (CNS) penetration, was evaluated in combination with bevacizumab (BEV).

**Methods:**

There were 2 phases, dose-escalation (3+3 design) and dose-expansion, which included patients (1) who progressed during or after first-line therapy (no prior BEV), (2) who progressed during or after second-line therapy with BEV, and (3) who had unresectable high-grade glioma (no prior BEV).

**Results:**

Sixty-four patients with high-grade glioma were treated; 18 in escalation cohorts and 46 in expansion Cohorts A (21), B (15), and C (10). The maximum-tolerated dose (MTD) was not reached and the RP2D was 400 mg capmatinib PO BID (800 mg daily). Treatment continued for a median of 14 weeks and up to ~6 years in one patient. Common treatment-related adverse events (65% ≤ Grade 2) included fatigue, peripheral edema, nausea, diarrhea, ALT increased, and constipation. Headaches and seizures occurred in 11 patients; Grade 3+ events included Grade 3 headache (1) and Grade 3 seizures (4). There were no treatment-related deaths. The 12 responders to treatment (2 CRs [1 pt in escalation and 1 pt in Cohort A] and 10 PRs [2 pts in escalation and A = 6, B = 1, and C = 1]) had a median duration of response of 9.2 months. Two patients with durable responses (CR >5 years, PR >1 year) did not harbor baseline c-MET alterations.

**Conclusion:**

Capmatinib + BEV was well-tolerated but had no clear signal of activity in c-MET non-activated high-grade glioma.

## Lay Summary

High-grade gliomas are fast-growing and aggressive brain tumors, and treatment options for them are limited. Capmatinib is a drug that targets a specific protein that can contribute to tumor growth. The authors of this study wanted to see if treatment with capmatinib can be helpful for patients with high-grade gliomas. To test this, they studied the use of capmatinib in combination with another drug called bevacizumab in a clinical trial with 64 patients who had high-grade gliomas. The authors found that the treatment was generally safe. Twelve patients had some shrinkage of their tumors. However, the treatment did not seem to help patients without high levels of protein being targeted.

**Key Points**

- Capmatinib was evaluated with bevacizumab in patients with high-grade glioma.
- Treatment was well-tolerated, with limited CNS events.
- No clear signal of activity in c-MET non-activated high-grade glioma was seen.

**Importance of the Study**

Capmatinib was not previously studied with bevacizumab in patients with high-grade glioma. This combination was investigated in this study to determine whether deeper and more durable responses could be observed with downstream inhibition of c-MET angiogenic resistance pathways compared to inhibition of VEGF pathways alone. Sixty-four patients with high-grade glioma were treated: 18 in escalation cohorts and 46 in expansion cohorts. The MTD was not reached and the RP2D was 400 mg capmatinib PO BID (800 mg daily). Treatment was well-tolerated with no deaths, and the most common events were fatigue, peripheral edema, nausea, diarrhea, ALT increased, and constipation. The 12 responders to treatment (2 CRs and 10 PRs) had a median duration of response of 9.2 months. While a clear signal of activity in c-MET non-activated high-grade glioma was not observed, future trials designed with more stringent biological and molecular criteria may show different results using this combination.

Despite the development of improved therapies for cancer patients in recent years, the prognosis for patients with recurrent high-grade glioma remains poor. After recurrence, further local or systemic treatments have little impact on survival.^1–5^ Gliomas are complex tumors that require a diverse array of downstream signaling for oncogenesis, such as epidermal growth factor (EGFR), vascular endothelial growth factor (VEGF), and others. c-MET signalization is associated with cell motility, invasion, and metastasis as well as mediates secondary resistance to commonly used targeted therapies against common pathways, such as EGFR or VEGF. Response rates with bevacizumab (BEV),^6^ a monoclonal antibody against circulating VEGF, range from 36% to 59% and progression-free survival (PFS) rates at 6 months (PFS-6) are estimated to be 36% to 51%.^7,8^ However, overall survival does not appear to increase in the first- or second-line setting in patients with gliomas.^9–11^ Other VEGF-targeting agents (pazopanib, cediranib, and aflibercept) have also been ineffective in extending survival.^12–14^ A lack of improvement in survival may be explained in part by the complex interaction of HGF (HIF-1a)/c-MET with VEGF and its receptors and their role during invasion, growth, and motility. c-MET upregulation itself may be a key factor contributing to resistance to angiogenic agents in gliomas.^15,16^ HIF-1a may accomplish this at least in part by recruiting bone marrow derived cells that in turn express matrix metalloproteinase 9 (MMP-9).^17^ MMP-9 is a critical downstream factor of HIF-1a that enables the angiogenic switch by making sequestered VEGF bioavailable for its receptor VEGFR2.^18–20^ Similarly, MMP-9 deficiency impedes neovascularization in glioblastoma due to the inability of VEGF to bind to its receptor.^21^

To improve treatment efficacy, this study evaluated INC280 (capmatinib), a c-MET inhibitor, with BEV. Capmatinib is a highly selective and potent oral inhibitor of MET receptor with robust central nervous system (CNS) penetration that is approved by the US FDA for treatment of NSCLC-harboring MET exon 14 skipping alterations.^22^ By selectively binding to c-MET and inhibiting c-MET phosphorylation, c-MET signal transduction pathways are disrupted inducing cell death in tumor cells overexpressing c-MET protein or expressing constitutively activated c-MET protein.^23^ Our hypothesis is that by inhibiting downstream HIF1-a/c-MET angiogenic resistance pathways, deeper and more durable responses would be observed compared to inhibition of VEGF pathways alone.

## Patients and Methods

This study was conducted by the Sarah Cannon Research Institute (SCRI) at 5 centers in the United States according to the ethical principles of the International Council for Harmonisation Guidance. The institutional review boards of all participating sites approved the study and patients were enrolled following written informed consent. This trial is registered with ClinicalTrials.gov (NCT02386826).

### Study Design

This open-label, non-randomized Phase Ib study was designed to determine the maximum-tolerated dose (MTD) of capmatinib to be administered in combination with BEV as treatment for patients with advanced high-grade glioma. The trial included 2 phases, a dose-escalation phase with increasing doses of oral capmatinib combined with standard doses of BEV to determine the recommended Phase 2 dose (RP2D) and an expansion phase in 3 cohorts of patients with high-grade glioma at the RP2D. Cohort included (1) those who progressed during or after first-line therapy (no prior BEV); (2) those who progressed during or after second-line therapy with BEV; and (3) those who had unresectable high-grade glioma and no prior BEV. Patients in Cohorts A and B were assessed for response by MRI using Response Assessment in Neuro-Oncology (RANO) criteria^24^ every 2 cycles (every 8 weeks). For patients in Cohort C, restaging occurred after the first cycle of neoadjuvant therapy to assess response to treatment and feasibility of surgical resection. The pharmacokinetics of the combination were assessed in 10 patients in Cohort A.

### Eligibility

Eligible patients had a histologic diagnosis of high-grade glioma as defined by the 2016 World Health Organization (WHO) classification system, that is, patients with glioblastoma, gliosarcoma, and Grade 4 anaplastic astrocytoma. This classification system allowed a patient with glioblastoma to be included in the study even though their disease was considered Grade 1. Since the trial was conceived prior to the 2021 classification and approved by the Institutional Review Board at the time of study initiation, data was only analyzed according to the approved protocol. However, a post-hoc analysis including retrospective analysis of molecular classification is currently underway. Patients that progressed during or after standard first-line therapy were eligible for the escalation phase, and for Cohort A of the expansion phase. This included patients scheduled to undergo a repeat primary surgical resection. For Cohort B of expansion, patients had a histologic diagnosis of high-grade glioma, and were to have progressed on at least 2 prior lines of therapy with the most recent therapy including BEV or a BEV-based regimen. For Cohort C of expansion, patients had first-line unresectable brain tumors and a histologic diagnosis of high-grade glioma by stereotactic biopsy and were treated with neoadjuvant intent. Previous treatment with BEV was not allowed for patients in Cohorts A or C.

Additional entry criteria for both phases of the study included the following: age ≥18 years, Eastern Cooperative Oncology Group (ECOG) performance status of 0 to 2, and adequate hematologic, liver, and renal function. Patients receiving drugs known to be strong inhibitors or inducers of cytochrome P450 (CYP) 3A4 (CYP3A4) that could not be discontinued 7 days before the start of capmatinib were not allowed.

### Treatment Regimen

The first patient cohort in dose-escalation received capmatinib 200 mg PO (100 mg twice daily) on a 28-day cycle in combination with BEV 10 mg/kg IV every 2 weeks. The dose of capmatinib escalated to 400 mg PO daily (200 mg twice daily), and then to 800 mg PO daily (400 mg twice daily) in subsequent cohorts of 3 to 6 patients who all received BEV 10 mg/kg IV every 2 weeks. All patients in the expansion phases received the RP2D every 2 weeks on a 28-day cycle except patients in Cohort C (unresectable high-grade glioma) who received 800 mg capmatinib in combination with 15 mg/kg IV BEV every 4 weeks for 1 cycle of treatment. For Cohort C, neurologically stable patients with complete or partial responses after one cycle of treatment, and whose tumor was now resectable, were to undergo surgical resection, chemotherapy, or radiation therapy per standard of care (SOC). If the patient’s tumor was still unresectable, they could receive another cycle of treatment with neoadjuvant capmatinib+15 mg/kg BEV and undergo a repeat MRI after 28 days of treatment. If the patient’s tumor was still not resectable after 2 cycles of treatment, definitive chemoradiation therapy or surgery per SOC were considered.

### Dose Modiﬁcations

While no dose reductions of BEV were allowed, up to 2 dose reductions of capmatinib were allowed for toxicity. Protocol defined dose-limiting toxicities (DLTs) included Grade 3 hematologic toxicities lasting for >4 consecutive days or ≥ Grade 4 (of any duration); febrile neutropenia; renal, hepatic, pancreatic, and cardiac toxicities ≥ Grade 3; neurologic toxicities ≥ Grade 2 lasting for >4 days; and any toxicity that led to delay or interruption of treatment for >3 weeks. The minimum dose of capmatinib permitted was 200 mg (as a divided dose of 100 mg twice per day). If treatment with either drug was delayed for >3 weeks due to toxicity, the offending study medication was discontinued. If 1 drug was held due to toxicity, treatment with the other drug was allowed to continue, as appropriate. If BEV was held due to toxicity, the dose remained the same once treatment resumed. Specific management was detailed in the protocol for anticipated hematologic capmatinib-related toxicities. Bevacizumab-related toxicity was managed according to standard medical practice.

### Deﬁnition of Response

Patients in the expansion groups (Cohorts A and B) were evaluated for response to treatment after 2 cycles (8-week intervals ±1 week) of treatment and were restaged per RANO criteria.^24^ Patients with progressive disease (PD) or unacceptable toxicity were discontinued from the study; patients with stable disease (SD) or response to therapy continued treatment.

### Correlative Analyses

Archival formalin-fixed paraffin-embedded tumor biopsy samples were collected from all patients at study entry for biomarker testing if they were available. PathGroup evaluated the collected tumor tissue for c-MET (mesenchymal-epithelial transition factor) by immunohistochemistry (IHC) and c-MET amplification by fluorescence in situ hybridization (FISH). In addition, the tissue was sent to Foundation Medicine for molecular profiling using the Foundation One assay.^25^ For patients in Cohort C whose tumors became resectable after treatment with neoadjuvant capmatinib + BEV, fresh frozen tumor tissue was collected at surgery for biomarker testing. In addition, cerebrospinal fluid (CSF) was collected predose by lumbar puncture for PK analysis on Cycle 1 Day 15 from the first 3 patients enrolled to Cohort C who must have received capmatinib for at least 3 days prior.

### Pharmacokinetics

Pharmacokinetic blood samples were drawn from 10 patients in Cohort A at the following time points: Cycle 1 Day 15: predose, 2 h (±10 min) and 6 h (±30 min) post dose and Cycle 2, Day 1 predose.

### Statistical Analyses

For dose escalation, a maximum of 3 capmatinib dose levels (maximum 18 patients) were to be evaluated to determine the MTD using a standard 3 + 3 dose-escalation pattern. Response to treatment was evaluated in all patients using the RANO criteria. Approximately 63 patients were planned to be enrolled. This sample size was chosen to provide a preliminary safety and PK assessment of the combination of capmatinib + BEV.

The analysis of PK data was provided by WuXi AppTec, Laboratory Testing Division. Concentration–time data were analyzed with the NCA and Descriptive Stats modules in Pharsight WinNONLIN 7.0 validated using the Validation Suite provided by Pharsight/Certara.

## Results

### Patient and Disease Characteristics

Between September 2015 and March 2019, 64 patients were enrolled and treated in this study. Thirty-seven (57.8%) patients were male and 27 (42.2%) were female, with a median age of 58.5 years (range 26 to 77 years); 87.5% were White and 4.7% were Black or African American; 53 (82.8%) patients had baseline ECOG performance status of 0 or 1 (Table 1).

**Table 1.** Demographic and Disease Characteristics (*N* = 64)

Age (years) at enrollment	*N* (%)
Median (min, max)	58.5 (26, 77)
**Sex**	
Male	37 (57.8)
Female	27 (42.2)
**Race**	
White	56 (87.5)
Black or African American	3 (4.7)
Other	5 (7.8)
**Pretreatment ECOG**	
0	16 (25.0)
1	37 (57.8)
2	11 (17.2)
**Histology**	
Glioblastoma	64 (100.0%)
**Histology Grade (G)**	
G1	1 (1.6%)
G4	59 (92.2%)
GX	4 (6.3%)
**Brain involvement at study entry**	
Brain only, single site	41 (64.1%)
Brain only, multifocal	23 (35.9%)
**Prior resection**	
Yes	53 (82.8%)
No	11 (17.2%)
**Lateralization**	
Right	29 (45.3%)
Left	28 (43.8%)
Bilateral	5 (7.8%)
Unknown	2 (3.1%)

ECOG = Eastern Cooperative Oncology Group (Performance Status); GX = the grade could not be assessed.

High-grade glioma was the primary diagnosis for all patients (Grade 1 = 1 [1.6%]; Grade 4 = 59 [92.2%]; and 4 [6.3%] designated as Grade X because the grade could not be determined). Fifty-three (82.8%) patients had undergone previous surgery, 56 (87.5%) had received prior systemic therapy, and 56 (87.5%) had received prior radiotherapy. For their first surgical resection, 6 (9.4%) patients had complete resections and 47 (73.4%) had partial surgical resections.

### Disposition and Treatments Received

At the data cutoff of 21 July 2021, 1 patient was continuing treatment, and 63 patients were off study. Patients were on study for a median of 3.25 (0.4, 71.7) months. Six (9.4%) patients were treated in each of the 3 dose-escalation cohorts. No DLTs were observed during dose escalation and the MTD was not reached; the RP2D was determined to be 400 mg capmatinib PO BID (800 mg daily). All patients in the expansion cohorts received the RP2D; Cohort A: 21 (32.8%), Cohort B: 15 (23.4%), and Cohort C: 10 (15.6%). The main reasons for discontinuation of capmatinib and BEV were PD and study completion (Table 2). The 4 deaths on study were due to adverse events (AEs) of sepsis in 1 patient and diverticulitis in 1 patient both considered unrelated to either study drug or PD in 2 patients (Table 2).

**Table 2.** Patient Disposition—All Enrolled Patients (*N* = 64)

Subjects enrolled	64
Safety analysis set	64 (100.0%)
Efficacy-evaluable analysis set^a^	46 (71.9%)
**Treatment status**	
Patients still on treatment (at data cutoff)	1 (1.6%)
Patients off treatment	63 (98.4%)
**Patient deaths**	
Death due to disease	13 (20.3%)
Death due to AE	2 (3.1%)
Sepsis (unrelated, 1 pt)	
Diverticulitis (unrelated, 1 pt)	
**Reason for capmatinib treatment discontinuation**	
Progressive disease	48 (75.0%)
Completed	6 (9.4%)
Death^b^	4 (6.3%)
Adverse event^c^	3 (4.7%)
noncompliance with study drug	1 (1.6%)
Subject decision to discontinue treatment	1 (1.6%)
**Reason for BEV treatment discontinuation**	
Progressive disease	47 (73.4%)
Completed	6 (9.4%)
Adverse event^d^	4 (6.3%)
Death^b^	4 (6.3%)
noncompliance with study drug	1 (1.6%)
Subject decision to discontinue treatment	1 (1.6%)
**Cohort summary** ^e^	
Dose-escalation Cohorts 1, 2, and 3 each enrolled—MTD was not reached	18 (28.1%)
Dose expansion—Cohort A—Progressed during or after 1st line therapy	21 (32.8%)
Dose expansion—Cohort B—Progressed during or after 2nd line therapy with BEV	15 (23.4%)
Dose expansion—Cohort C—Patients with unresectable high-grade glioma	10 (15.6%)

^a^Evaluable population was defined as all patients who had received at least 1 cycle of study treatment at the RP2D and who had at least 1 postbaseline tumor assessment.

^b^Deaths were due to sepsis (unrelated to either drug, 1 pt), diverticulitis (unrelated to either drug, 1 pt), and PD (2 pts).

^c^Alanine aminotransferase increased (1 pt), infusion reaction (1 pt), peripheral edema (1 pt).

^d^Alanine aminotransferase increased (1 pt), hypertension (1 pt), peripheral edema (1 pt), infusion reaction to capmatinib (1 pt).

^e^All expansion cohorts received RP2D dose of capmatinib at 400 mg PO BID.

AE = adverse event; BEV = bevacizumab; BID = twice daily; MTD = Maximum-tolerated dose; PO = *per os* (orally); RP2D = recommended Phase 2 dose.

Patients received treatment with both study drugs for a median of 14 weeks, and 1 patient continued treatment for almost 6 years (311.6 weeks). Few patients required dose reductions of capmatinib (7 [10.9%] required 1 reduction and 2 patients [3.13%] required either 2 or 3 reductions). Dose interruptions of capmatinib occurred for 37 (58.7%) patients and interruptions of BEV for 22 (34.4%) patients, mainly due to AEs (Table 3).

**Table 3.** Treatment Duration, Exposure, and Modification (*N* = 63)

Duration on treatment (weeks)^a^	Capmatinib (*N* = 63)	Bevacizumab (*N* = 64)
Mean	25.6	25.3
Median (range)	14.1 (1.9, 311.6)	14.1 (1.9, 311.6)
**Dose reductions**	***N* (%)**	***N* (%)**
Patients with none	52 (81.2%)	64 (100.00%)
Patients with 1	7 (10.9%)	-
Patients with 2	2 (3.1%)	-
Patients with 3 or more	2 (3.1%)	-
**Reason for reduction**		
Adverse event^b^	8 (12.5%)	NA
Dosing error	2 (3.1%)	NA
Other	2 (3.1%)	NA
**Dose interruptions**		
Patients with none	26 (40.6%)	42 (65.6%)
Patients with 1	19 (29.7%)	8 (12.5%)
Patients with 2	10 (15.6%)	6 (9.4%)
Patients with 3 or more	8 (12.5%)	8 (12.5%)
**Reason for interruption**		
Adverse event	31 (48.4%)	19 (29.7%)
Dosing error	1 (1.6%)	5 (7.8%)
Other	10 (15.6%)	3 (4.7%)

^a^Two patients remained on single agent capmatinib after discontinuation of BEV for 18 and 52 additional weeks.

^b^ALT increased (3 patients [pts]), creatinine increased (2 pts), lymphedema (1 pt), peripheral edema and rash, (1 pt), dehydration and pruritis (1 pt).

### Correlative Studies

#### c-MET expression by IHC and c-MET amplification by FISH

Thirty-three samples from Cohorts A, B, and C (17, 9, and 7, respectively) were analyzed for c-MET expression and c-MET amplification; 2 patients (1 each in Cohorts A and C) were positive for both c-MET expression and amplification but neither had an overall response of complete response (CR) or partial response (PR) per RANO. However, 3 patients in Cohort A and 1 patient in Cohort C were positive for c-MET amplification with 1 of the Cohort A patients having a PR per RANO.

All 6 samples from the escalation DL3 (RP2D) cohort were analyzed for c-MET expression and c-MET amplification. Three of the 6 patients (50%) had response to treatment per RANO (1 CR, 2 PR). One of the patients with a PR was weakly positive for c-MET expression by IHC and positive for c-MET amplification by FISH, while the other patient with a PR and the patient with a CR were both negative for c-MET expression and amplification.

#### Foundation medicine molecular profiling

Thirty-one (31) samples were analyzed for molecular profiling including 4 from the dose-escalation DL3 cohort and 27 from the dose-expansion Cohorts A, B, and C (14, 6, and 7, respectively). For the DL3 cohort, results were available for only 1 of the responders who had a PR (short variant, PREX2 gene).

In Cohort A, 5 patients presented with EGFR, 3 patients with IDH1 mutations, and 1 patient with c-MET alterations. In Cohort B, 2 patients presented with EGFR, 2 patients with PIK3CA, and 1 patient with KRAS alterations. In Cohort C, 4 patients presented with EGFR, 2 patients with c-MET (short variant, copy number alteration), and 1 patient with PIK3CA alterations.

### Pharmacokinetics

#### Pharmacokinetic summary (first 10 Cohort A patients)

Since the time concentration data are sparse, PK analysis was limited. Plasma concentrations increased after dose, then decreased after 2 h post dose in 9 of 10 patients. Differences between the Cycle 1 and Cycle 2 predose values increased in 4 of 10 patients with the rest roughly the same or decreasing. No pattern was obvious. *C*_max_ values ranged from 633 to 6,220 ng/mL: AUC values ranged from 2,490 to 22,000 ng·h/mL. These *C*_max_ and AUC values were in range with the values reported by Bang et al.^26^ in patients with advanced solid tumors.

#### Cerebrospinal fluid results (first 3 Cohort C patients)—

Capmatinib was detected in the CSF drawn from 3 patients in Cohort C. Concentrations ranged from 7.51 to 123 ng/mL following a minimum of 3 doses.

### Safety

Grade 3 or higher treatment-emergent AEs were reported in 19 (29.7%) patients including 10 (15.6%) Grade 3 or higher CNS events. There were no deaths considered related to the study drugs (Table 2). The most reported treatment-related AEs included fatigue 24 (37.5%), peripheral edema 16 (25.0%), nausea 15 (23.4%), diarrhea 12 (18.8%), ALT increased 7 (10.9%), and constipation 7 (10.9%). Headaches and seizures were each reported in 11 patients, regardless of the relationship to study drugs (1 Grade 3 headache and 4 Grade 3 seizures) (Table 4). Related Grade 3 serious AEs each reported in 1 patient included cerebrovascular accident, diarrhea, peripheral edema, lymphedema, pulmonary embolism, rash, embolism, and wound infection. The single Grade 4 related serious adverse event was alanine aminotransferase increased.

**Table 4.** Treatment-Related and CNS Adverse Events in >10% of Patients (*N* = 64)

	Grade 1 *N* (%)	Grade 2 *N* (%)	Grade 3 *N* (%)	Grade 4 *N* (%)	Total *N* (%)
**Any treatment-related adverse events**	**16 (25)**	**20 (31.2)**	**16 (25)**	**3 (4.7)**	**55 (85.9)**
Fatigue	15 (23.4)	9 (14.1)			24 (37.5)
Peripheral edema	8 (12.5)	7 (10.9)	1 (1.6)		16 (25)
Nausea	11 (17.2)	4 (6.2)			15 (23.4)
Diarrhea	10 (15.6)	1 (1.6)	1 (1.6)		12 (18.8)
ALT increased	1 (1.6)	1 (1.6)	3 (4.7)	2 (3.1)	7 (10.9)
Constipation	6 (9.4)	1 (1.6)			7 (10.9)
					
**Any CNS adverse events**	**23 (35.9)**	**12 (18.8)**	**10 (15.6)**		**45 (70.3)**
Headache	9 (14.1)	1 (1.6)	1 (1.6)		11 (17.2)
Seizure	4 (6.2)	3 (4.7)	4 (6.2)		11 (17.2)

Cerebrovascular accident (1 patient [pt]), diarrhea (1 pt), peripheral edema (1 pt), lymphedema (1 pt), pulmonary embolism (1 pt), rash (1 pt), embolism (1 pt), wound infection (1 pt).

### Efficacy

All patients in the expansion cohorts received the RP2D. There were 46 (72%) patients who were evaluable for response. Of the 18 patients considered not evaluable for response, 12 were from escalation cohorts DL1 and DL2 and received less than the RP2D and 6 patients were from Cohorts A, B, or C and did not meet the criteria for the evaluable population primarily due to PD before first scans on treatment. There were a total of 12 responders (Table 5).

**Table 5.** Response and Progression-free Survival Summary

	DL3 (RP2D) (*N* = 6), *N* (%)	Cohort A (*N* = 20), *N* (%)	Cohort B (*N* = 12), *N* (%)	Cohort C (*N* = 8), *N* (%)	Total (*N* = 46), *N* (%)
**Best overall response**					
Complete response (CR)	1 (16.7)	1 (5.0)			2 (4.3)
Partial response (PR)	2 (33.3)	6 (30.0)	1 (8.3)	1 (12.5)	10 (21.7)
Stable disease (SD)	3 (50.0)	7 (35.0)	2 (16.7)	5 (62.5)	17 (37.0)
Progressive disease (PD)		6 (30.0)	9 (75.0)	2 (25.0)	17 (37.0)
**Objective response rate (CR+PR) (ORR)**	3 (50.0)	7 (35.0)	1 (8.3)	1 (12.5)	12 (26.1)
95% exact binomial confidence interval (CI)					26.1% (14.3%, 41.1%)
95% asymptotic normal approximation CI					26.1% (13.4%, 38.8%)
**Median PFS (months) (95% CI) (range)**	7.3 (1.8, N/A)	4.1 (1.8, 5.6)	1.8 (1.2, 3.6)	N/A (1.1, N/A)	3.6 (1.9, 5.1)
**PFS probability at:**					
6 months (95% CI) (range)	50 (11.1, 80.4)	15.0 (3.7, 33.5)	18.2 (2.9, 44.2)	N/A	21.8 (10.4, 36.0)
12 months (95% CI) (range)	33.3 (4.6, 67.6)	10.0 (1.7, 27.2)	9.1 (0.5, 33.3)		13.7 (5.0, 26.5)
18 months (95% CI) (range)	N/A	10.0 (1.7, 27.2)	9.1 (0.5, 33.3)		10.2 (3.0, 22.8)
Correlative data	1 PR: MET amplification 1 CR & 1 PR: Polysomy of Chr 7	1 PR: MET amplification 1CR & 2 PRs: Polysomy of Chr 7 3 PR: no correlative data	1 PR: no correlative data	1PR: no correlative data	

The response and PFS summaries are based on the Evaluable Analysis Set.

◦Evaluable population is defined as all patients who received at least 1 cycle of study treatment at the RP2D and had at least 1 postbaseline tumor assessment.

◦Patients not included in the evaluable population included:

•12 patients treated below the RP2D in Dose Levels 1 and 2.

•6 patients in the expansion Cohorts A through C who did not meet the criteria for evaluable population due to PD (2 pts), death (3 pts), and participant decision to discontinue treatment (1 pt).

**Figure 1.**
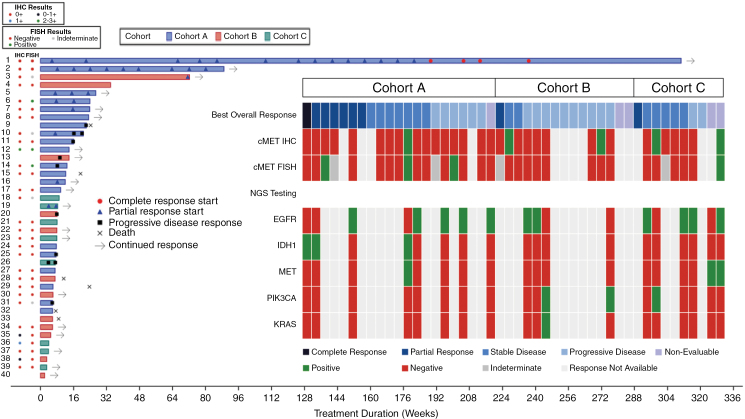
Swimmer’s plot of treatment duration and response in expansion and metabolic profile summary vs response. Notes: The Evaluable Analysis Set = all patients who received at least 1 cycle of study treatment at the RP2D and at least 1 postbaseline tumor assessment. Duration of treatment = (max [date of BEV discontinuation, date of capmatinib discontinuation]—[date when BEV started, date when capmatinib started] + 1) /7.

The objective response rate (CR+PR) was 26.1% (12 patients) with an exact binomial confidence interval of 26.1% (14.3%, 41.1%). Overall median PFS for the 46 evaluable patients was 3.6 months (95% CI 1.9, 5.1) with a 6-month probability of 21.8% (10.4, 36.0). The PFS probabilities at 6 months for the individual cohorts were: DL3 of escalation—50% (11.1, 80.4); Cohort A—15.0% (3.7, 33.5); and Cohort B—18.2% (2.9, 44.2) (Table 5). Figure 1 presents a swimmer’s plot of treatment duration and response.

Of the 12 patients with CR or PR responses, 9 had IHC/FISH results available. In the escalation DL3 cohort, 1 of the 2 patients with a PR was weakly positive for c-MET expression and positive for c-MET amplification, the other was not, and 4 patients were positive for c-MET amplification in Cohorts A (3) and C (1) combined, with 1 of the Cohort A patients having a PR per RANO. Radiographic responses and PFS of the 3 patients in Cohort A with known IDH mutations included 1 CR/PFS: 71.7 months; 1 PR/PFS: 20 months; and 1 SD/PFS: 3 months.

## Discussion

To our knowledge, this is the first trial designed to investigate the administration of capmatinib in combination with BEV as treatment for patients with advanced high-grade glioma. The combination of capmatinib and BEV was well-tolerated in most patients and there were no treatment-related deaths. The incidence and severity of reported events were consistent with the known toxicity profiles of capmatinib^22^ and BEV,^6^ both as single agents and when used in combination, except for a higher incidence of seizures (11 [17.2%]; Grades 1 to 3) than previously reported.

The median PFS of 3.6 months with BEV+capmatinib in all evaluable patients was comparable to that seen with BEV in combination with irinotecan or lomustine.^8,11^ The ORR was higher in the BEV-naïve patients in this study (50% and 35%) compared to patients in Cohort B who had received prior BEV (8.3%) and are comparable to response rates from previous studies with BEV+irinotecan in BEV-naïve patients with recurrent glioblastoma.^7,8^ The 6-month PFS rates with this combination are lower than expected, even in the BEV-naïve patients suggesting that the observed responses were not very durable. Our original hypothesis was that patients without c-MET alterations may respond better than patients with c-MET alterations due to inhibition of rescue pathways. Due to the induction of HGF/HIF-1a as a resistance pathway to VEGF signaling, the addition of c-MET inhibitor may induce durable responses that are not typically seen with VEGF inhibition alone in tumors that already did not have c-MET pathways activated. Overall, our findings were not consistent with this hypothesis. However, it should be noted that the only 2 patients on study who experienced a durable response (CR >5 years, PR >1 year) did not bear c-MET alterations at baseline. Furthermore, only 2 patients had tumors positive for c-MET expression and neither responded to treatment (Figure 1).

## Study Limitations and Conclusion

Our study was limited by the inability to observe the extent of abrogation of downstream HIF1-a/c-MET angiogenic resistance pathways and whether this biologic correlate made clinical impact. Pre- and post-biopsies in this patient population are difficult to obtain but would have added scientific strength. In addition, details of post-surgery SOC therapy for the 2 patients in Cohort C who had surgery following neoadjuvant study treatment would have been of interest. These therapies were at the Investigator’s discretion, however, and were not recorded in the database. In conclusion, the combination of capmatinib with bevacizumab was well-tolerated but was without a clear signal of activity in c-MET non-activated high-grade glioma. However, future trials designed with more stringent biological and molecular criteria may show different results using this combination.

## Data Availability

The data for this document will be made available upon reasonable request.
